# Genetic Features of Extended-Spectrum β-Lactamase-Producing *Escherichia coli* from Poultry in Mayabeque Province, Cuba

**DOI:** 10.3390/antibiotics10020107

**Published:** 2021-01-22

**Authors:** Michel Baez, Ivette Espinosa, Alexandra Collaud, Iliana Miranda, Damarys de las Nieves Montano, Angel L. Feria, Rosa Elena Hernández-Fillor, Dasiel Obregón, Pastor Alfonso, Vincent Perreten

**Affiliations:** 1 National Center for Animal and Plant Health, Tapaste Road and National Highway, Postal Mail 10, San José de las Lajas, Mayabeque 32700, Cuba; michel@censa.edu.cu (M.B.); espinosa@censa.edu.cu (I.E.); ileanam@censa.edu.cu (I.M.); damarysmv@censa.edu.cu (D.d.l.N.M.); rosaelena25@censa.edu.cu (R.E.H.-F.); alfonso@censa.edu.cu (P.A.); 2 Institute of Veterinary Bacteriology, Vetsuisse Faculty, University of Bern, Länggassstrasse 122, 3012 Bern, Switzerland; alexandra.collaud@vetsuisse.unibe.ch; 3 Mayabeque Poultry Company, Ave 69, # 7210 between 72 and 74 Guines, Mayabeque 33900, Cuba; veterinario@avimay.geg.cu; 4 Center for Nuclear in Agriculture, University of São Paulo, Piracicaba, SP 13400-970, Brazil; dasiel@usp.br; 5 School of Environmental Sciences, University of Guelph, Guelph, ON N1G 2W1, Canada

**Keywords:** third-generation cephalosporins, ESBL, *Escherichia coli*, chicken, antibiotic resistance, genotyping

## Abstract

A total of 434 poultry cloacal samples were collected from seven different farms in different years (2013–2015) in the Cuban province of Mayabeque and analyzed for the presence of third-generation cephalosporin-resistant *Escherichia coli* (3GC-R-*Ec*). Sixty-two 3GC-R-*Ec* isolates were recovered in total from the farms, with detection rates of 2.9% in 2013, 10.3% in 2014, and 28.7% in 2015. Characterization of 32 3GC-R-*Ec* isolates revealed the presence of the extended-spectrum β-lactamase (ESBL) genes *bla*_CTX-M-1_ (*n* = 27), *bla*_CTX-M-15_ (*n* = 4), and *bla*_CTX-M-1_ together with *bla*_LAP-2_ (*n* = 1). The isolates also contained different proportions of genes conferring decreased susceptibility to sulfonamides (*sul1*, *sul2*, *sul3*), trimethoprim (*dfrA1*, *dfrA*7, *dfrA*12, *dfrA*14, *dfrA*17), tetracyclines (*tet*(A), *tet*(B)), aminoglycosides (*aac(6′)-Ib-cr*, *strA*, *strB*), chloramphenicol (*cmlA1*, *floR*), macrolides (*mph*(A), *mph*(D)), and quinolones (*qnrS*, *qnrB*, *aac(6′)-Ib-cr*) as well as mutations in the fluoroquinolone-resistance determining regions of GyrA (S83L, D87N, D87Y) and ParC (S80I, E84G). The isolates belonged to 23 different sequence types and to phylogroups A (*n* = 25), B1 (*n* = 5), and D (*n* = 2), and they contained plasmid-associated incompatibility groups FII, X1, HI1, HI2, N, FIA, and FIB. These findings reveal a genetically diverse population of multiresistant ESBL-producing *E. coli* in poultry farms in Cuba, which suggests multiple sources of contamination and the acquisition of antibiotic resistance genes.

## 1. Introduction

The overuse and misuse of antimicrobials in animals and humans have favored the spread of antibacterial resistance worldwide [1,2]. Strains of third-generation cephalosporin-resistant *E. coli* (3GC-R-*Ec*) in humans and food-producing animals have been widely and increasingly reported over the last decade [3,4]. Poultry plays an important role as a reservoir of 3GC-R-*Ec* in many countries around the world [5,6]. Third-generation cephalosporin resistance in *E. coli* is mainly mediated by the acquisition of genes encoding Ambler class A extended-spectrum β-lactamases (ESBLs) and class C plasmid-mediated AmpC (pAmpC) enzymes. The two main families of ESBLs in 3GC-R-*Ec* are the CTX-M and SHV types, whereas CMY-type enzymes are the most common pAmpC enzymes [7,8]. The CTX-M type appears to be the most widely disseminated group in animals and humans worldwide [9,10,11,12]. The ESBL and pAmpC genes are frequently co-located with other antibiotic resistances genes on conjugative plasmids which may be maintained and transferred within the *Enterobacterales* population by the use of different classes of antibiotics against infection diseases [5,6]. Such bacteria may reach the human gut via the food chain and food preparation, and their plasmids be transferred to human pathogens [3,5,6].

Strains of 3GC-R-*Ec* are also frequently resistant to fluoroquinolones, with resistance to the latter resulting from either mutations in the quinolone resistance-determining regions (QRDRs) of the chromosomal topoisomerases GyrA and ParC or the acquisition of plasmid-mediated quinolone resistance (PMQR) genes [13,14]. Multiple lines of evidence supported by experimental models and studies of genetic relationships indicate that some avian strains of *E. coli* may share similar pathogenic features to extraintestinal pathogenic *E. coli* (ExPEC) strains in humans [15,16]. In addition, associations between *E. coli* colonization and infection in humans and the exposure of *E*. *coli* to retail chicken and other food sources have been reported [17,18]. Animals, particularly poultry, carrying 3GC-R-*Ec* have raised major public health concerns, since the mobile genetic elements and antibiotic resistance genes associated with 3GC-R-*Ec* have been linked to human isolates that cause infection [19,20,21,22]. In addition, some studies have revealed the roles of highly epidemic clones of 3GC-R-*Ec* in the dissemination of antimicrobial-resistant genes among humans, animals and the environment [23,24]. For instance, *E. coli* belonging to sequence type ST410 and phylogroup A and containing the *bla*_CTX-M-15_ gene on plasmids of incompatibility (Inc) groups FIA and FIB has been recognized as a successful and epidemic clone spreading in animals, humans and the environment [23,25,26,27,28]. Additionally, epidemic-resistance plasmids belonging to group IncF with divergent replicon types (e.g., FIA, FIB and FII) have the ability to acquire resistance genes and rapidly disseminate among members of *Enterobacteriaceae*, especially among certain clones within species [29].

In Cuba, 3GC-R-*Ec* isolates have been identified in the environment and in pigs and humans, with the CTX-M genotype being predominant [28,30,31,32]. However, to date, the presence of 3GC-R-*Ec* in poultry has not been investigated. In the present study, poultry from poultry farms across the province of Mayabeque in Cuba were screened for the presence of 3GC-R-*Ec*, and a set of isolates were selected for antimicrobial susceptibility testing and characterization of their genetic background.

## 2. Results

### 2.1. Distribution of 3GC-R-Ec

**Figure 1:**
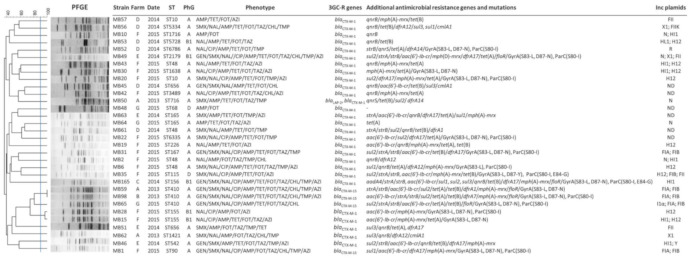
Genetic characteristics and phylogenetic tree constructed from the pulsed-field gel electrophoresis (PFGE) patterns of 32 3rd-generation cephalosporin-resistant *E. coli* (3GC-R-*Ec*) isolated from poultry in the province of Mayabeque, Cuba. Farms are represented by the letters A to G. ST: sequence type; PhG: phylogroup; Inc plasmids: incompatibility groups of plasmids. Antibiotics: AMP, ampicillin; CAZ, ceftazidime; CTX, cefotaxime; CIP, ciprofloxacin; NAL, nalidixic acid; GEN, gentamicin; SMX, sulfamethoxazole; TMP, trimethoprim; TET, tetracycline; CHL, chloramphenicol; AZI, azithromycin. Antimicrobial resistance genes and functions: *bla*_CTX-M_, extended-spectrum β-lactamase genes for resistance to 3rd-generation cephalosporins; *aadA4*, aminoglycoside nucleotidyltransferase gene for streptomycin and spectinomycin resistance; *cmlA1*, chloramphenicol efflux gene; *floR*, florfenicol/chloramphenicol resistance gene; *dfrA1, dfrA7, dfrA12, dfrA14, dfrA17*, and *drfA19*, dihydrofolate reductase genes for trimethoprim resistance; *tet*(A), *tet*(B), tetracycline efflux genes; *strA*, *strB*, streptomycin phosphotransferase genes; *sul1, sul2*, *sul3*, dihydropteroate synthase genes for sulfonamide resistance; *mph*(A)-*mrx*, *mph*(D)-*mrx*, macrolides inactivation gene cluster; *aac(6′)-Ib-cr*, aminoglycoside N(6′)-acetyltransferase-cr gene for amikacin, kanamycin and quinolone resistance; *qnrB*, *qnrS*, DNA gyrase protection genes for low level resistance to fluoroquinolones. GyrA (S83-L), (D87-N), (D87-Y) and ParC (S80-I), (E84-G), amino acid substitutions in topoisomerases GyrA and ParC for high-level resistance to fluoroquinolones.

Sixty-two 3GC-R-*Ec* isolates were obtained from the 434 cloacal samples collected at the seven different poultry farms (A–G) in Mayabeque Province, Cuba, between 2013 and 2015 (Figure 1). In 2013, 3GC-R-*Ec* isolates were detected at farms A and B (4/139; 2.9% (95% confidence interval (CI) 1.1–7.1%)); in 2014, they were detected at farms C, D, and E (15/145; 10.3% (95% CI 6.4–16.4%)); and in 2015, they were detected at farms F and G (43/150; 28.7% (95% CI 22–36.4%)). 3GC-R-*Ec* was detected at all investigated poultry farms but less frequently at the cage-laying hen farms (22/62 (35.5%) isolates, representing the six farms A–E and G) than at the ground-reared broiler farm (40/62 (64.5%) isolates, farm F).

Based on the rep-PCR profiles and origins of the 62 3GC-R-*Ec* isolates (Figure S1), 32 isolates were selected for antimicrobial susceptibility testing, molecular typing, and phylogenetic analysis. All 32 selected isolates were resistant to the third-generation cephalosporin cefotaxime, and 20 of them were also resistant to ceftazidime (Figure 1 and Table S1). In addition to exhibiting resistance to β-lactams, the majority of the isolates (*n* = 30) exhibited resistance to other antimicrobials, such as tetracycline (*n* = 26), sulfamethoxazole (*n* = 19), trimethoprim (*n* = 18), nalidixic acid (*n* = 24), ciprofloxacin (*n* = 16), chloramphenicol (*n* = 11) and gentamicin (*n* = 9). Fourteen isolates showed decreased susceptibility to azithromycin, with minimal inhibitory concentration (MIC) values above 16 mg/L. The isolates were susceptible to tigecycline, meropenem and colistin (Figure 1 and Table S1). 

Resistance to third-generation cephalosporins was associated with the presence of the ESBL genes *bla*_CTX-M-1_ (*n* = 28), *bla*_CTX-M-15_ (*n* = 4), and *bla*_LAP-2_ (*n* = 1) (Figure 1 and Table S1). Resistances to other antimicrobials were associated with genes conferring resistance to sulfonamides (*sul1* (*n* = 5), *sul2* (*n* = 12), *sul3* (*n* = 5)), trimethoprim (*dfrA*1 (*n* = 1), *dfrA*7 (*n* = 1), *dfrA*12 (*n* = 4), *dfrA*14 (*n* = 2), *dfrA*17 (*n* = 8)), tetracyclines (*tet*(A) (*n* = 16), *tet*(B) (*n* = 14)), and chloramphenicol (*cmlA1* (*n* = 3), *floR* (*n* = 5). High-level resistance to fluoroquinolones was associated with amino acid substitution in GyrA (S83-L (*n* = 15), D87-N (*n* = 13), D87-Y (*n* = 1)) and ParC (S80-I (*n* = 12), E84-G (*n* = 2)) (Figure 1 and Table S1). In addition, the *qnr* and *aac(6)-Ib-cr* genes, which confer low-level resistance to fluoroquinolones [13], were found in 17 and 15 isolates, respectively. Some of the isolates also contained the streptomycin resistance genes *strA* and *strB* (*n* = 8), the streptomycin/spectinomycin resistance gene *aadA4* (*n* = 1), and the macrolide resistance genes *mph*(A) (*n* = 16) and *mph*(D) (*n* = 1). No gentamicin-resistance genes detectable with the microarray were found among the gentamicin-resistant isolates. Similarly, no chloramphenicol-resistance genes were detected in the chloramphenicol-resistant isolates MB1, MB2, and MB42, which exhibited an MIC of 16 mg/L (Figure 1 and Table S1).

### 2.2. Genetic Diversity of the 3GC-R-Ec Isolates

Twenty-five of the *E. coli* isolates belonged to phylogenetic group A, 5 to B1, and 2 to D (Figure 1). The isolates were highly diverse, exhibiting 29 different PFGE patterns and belonging to 23 different STs, with ST48 (*n* = 4), ST410 (*n* = 3), ST155 (*n* = 2), ST165 (*n* = 2), ST656 (*n* = 2) and ST10 (*n* = 2) identified more than once; the remaining 17 isolates belonged to unique STs (Figure 1).

Similar PFGE patterns were generated for only two isolates belonging to ST410, obtained from two different farms (A, B), and for isolates of ST90 and ST155, obtained from the same farm (F). The other isolates of the same ST exhibited different PFGE patterns. All the isolates of a given ST were from different farms except for two *E. coli* isolates of ST48, which were from the same farm (Figure 1).

**Figure 2:**
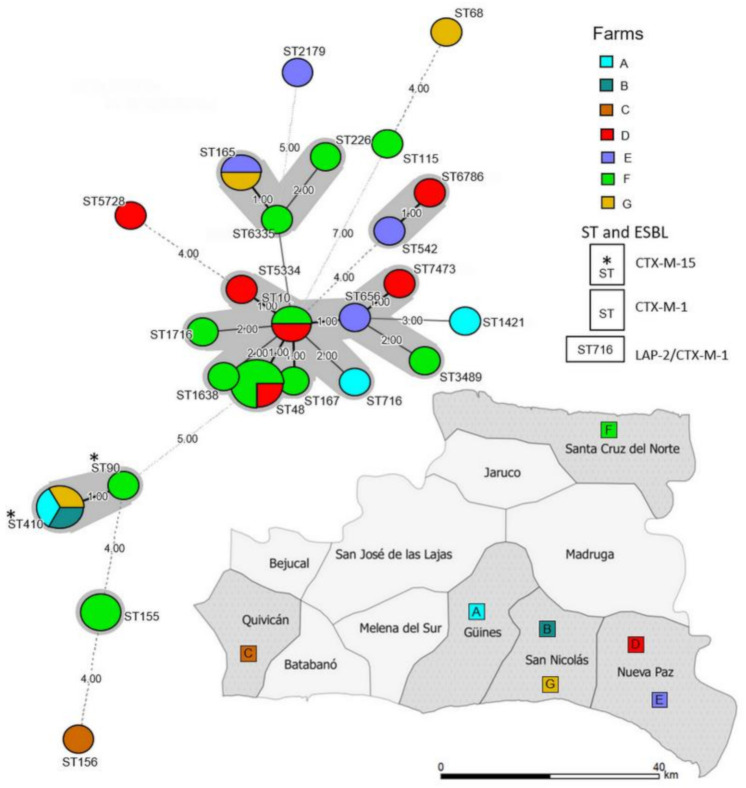
Minimum spanning tree of 3rd-generation cephalosporin-resistant *E. coli* (3GC-R-*Ec*) isolates from poultry farms in the province of Mayabeque, Cuba. Each circle represents one sequence type (ST). Lines between the circles indicate relationships between the different STs. Gray color around the circles indicates that the isolates share at least 5 alleles. The color of each circle corresponds to the color of the farm (A–G) where the isolates came from. An asterisk (*) next to an ST indicates that the isolates contained a CTX-M-15 β-lactamase. The absence of an asterisk next to an ST indicates that the isolate contained a CTX-M-1 β-lactamase. *E. coli* ST716 contained a LAP-2 β-lactamase. The geographical locations of the farms are represented on the map with the same color denoting the farms in the tree.

The isolates harboring the predominant ESBL, CTX-M-1, were highly diverse, exhibited diverse PFGE profiles, belonged to different STs and harbored plasmids of several different Inc groups. They were distributed across the six farms. In contrast, the isolates containing CTX-M-15 belonged to isolates of only two phylogenetically related STs, namely, ST410 and ST90. However, they were obtained from different farms (Figure 1 and Figure 2).

**Figure 3:**
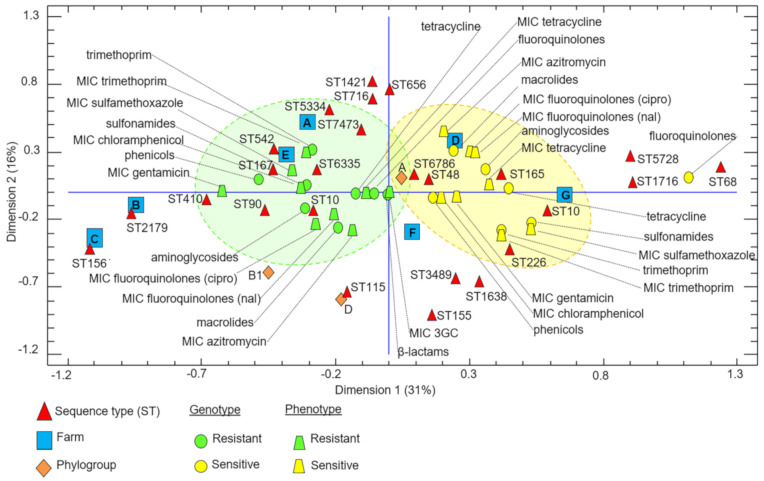
Multiple correspondence plot (2D) showing associations between the sequence type (ST) of the 3rd-generation cephalosporin-resistant *E. coli* isolates from poultry in the province of Mayabeque, Cuba. The multiple correspondence analysis (MCA) was based on genotypic and phenotypic characteristics of resistance or susceptibility to different classes of antibiotics as well as classification by phylogroup and the distributions of the isolates among the seven poultry farms. Abbreviations: nal, nalidixic acid; cipro, ciprofloxacin; 3GC, 3rd-generation cephalosporin. The green dotted ellipsoid contains a group of isolates with major patterns of resistance to non-β-lactam antibiotics. The yellow ellipsoid contains a group of isolates with higher susceptibility to non-β-lactam antibiotics. The fraction of each component is represented as a percentage for each dimension (axis).

The multiple correspondence analysis (MCA) revealed patterns of similarity among some of the isolates, which were based mainly on their genotype and phenotype of resistance or susceptibility to antibiotics (Figure 3). This analysis also revealed an association between antimicrobial resistance pattern and the geographic location of the isolates. For instance, all multiresistant isolates harboring CTX-M-15 and some harboring CTX-M-1 belonged to the STs/phylogroups ST10/A, ST156/B1, ST167/A and ST115/D and were most commonly found at farms A, B, C and E. In contrast, isolates exhibiting less resistance (i.e., those belonging to ST68/D, ST1716/A, ST155/B1 and ST165/A) were predominantly found at farms D, F and G. The MCA also indicated that combined resistance to 3GCs, fluoroquinolones and tetracycline is a common resistance trait for most of the *E. coli* strains from poultry in the region of Mayabeque.

## 3. Discussion

This study provides the first insight into the distribution and genetic features of 3GC-R-*Ec* in poultry in Cuba. Although the sampling was performed at different farms each year, the percentage of detected ESBL-producing *E. coli* isolates increased each year, increasing from 2.9% in 2013 to 28.7% in 2015. These results are inconclusive but are consistent with the epidemiological situation of 3GC-R-*Ec* worldwide, where an increase in the prevalence of ESBL-producing *E. coli* has been observed in both animals and humans [3,24,33]. The high frequency of 3GC-R-*Ec* at some of the investigated farms highlights the need for nationwide monitoring of antibiotic resistance in food-producing animals in Cuba, as has been established in several other countries [34,35].

The phylogenetic group A was the most common group, followed by phylogenetic group B1. The phylogenetic groups A and B1 generally do not include pathogenic *E. coli* and have been reported to be more common in animals than in humans in tropical areas [36,37]. Two isolates belonged to phylogenetic group D; this group, together with group B2, is associated with ExPEC infections in humans [38]. Nevertheless, in recent years, a successful clone of ST410 belonging to phylogenetic group A and disseminated worldwide in animals and environments has been found to be associated with ExPEC infections in humans [23,26,37,39,40]. The detection of such a 3GC-R-*Ec* clone in different farms and in different years underlines the possible spread and persistence of multiresistant isolates with pathogenic potential in poultry production and emphasizes the importance of implementing a One Health-based surveillance system for antibiotic-resistant bacteria. Indeed, the mechanism of transmission of β-lactamases in poultry farms in Cuba is not well elucidated, and multiple sources of contamination need to be considered, such as various environments, the flies and feces of other animals (i.e., rats, wild birds, dogs, cats, and other food-producing animals), and poultry workers [11,41,42].

In addition to exhibiting resistance to 3rd-generation cephalosporins, the majority of the isolates exhibited resistance to other classes of antibiotics, as has been observed for *E. coli* from poultry in other regions around the world [6,43,44,45]. In our study, the most common trait associated with resistance to third-generation cephalosporins was the presence of the CTX-M-1 ESBL. This enzyme is also the most common ESBL type in *E. coli* from poultry in regions worldwide [6,46] other than South American regions, where CTX-M-2, CTX-M-8, CTX-M-65, CTX-M-55 and CTX-M-3 are the predominant ESBLs in avian *E. coli* [44,45,47,48]. The isolates of ST410 in this study contained CTX-M-15, which is the predominant ESBL type in human *E. coli* worldwide [49]. Both CTX-M-1 and CTX-M-15 have been detected in 3GC-R-*Ec* isolates from patients of Cuban hospitals [28,32]. Nevertheless, the genetic relatedness between isolates and the CTX-M-containing genetic elements has not yet been investigated in Cuba; such investigation would require the whole genome-based characterization of bacterial isolates and large-scale epidemiological information [50,51]. However, our study showed that only the *E. coli* isolates of ST410 shared the same STs, phylogenetic groups and CTX-M determinant (ST410/A/CTX-M-15) as clinical *E. coli* isolates obtained from hospitalized patients in Cuba [28]. In addition, *E. coli* of ST10/A and ST167/A has been found in both poultry and humans in Cuba. However, the poultry isolates contained CTX-M-1, whereas the human isolates contained CTX-M-15 [28].

The poultry *E. coli* isolates from Cuba belonged to several different STs, some of which (e.g., ST10, ST48, ST155, ST68, ST542, ST226, ST656) have also been identified among *E. coli* isolates from chickens in Europe and Africa [3,11,52]. In Latin America, the STs reported for *E. coli* from poultry comprise ST113, ST130, ST131 and ST132 (Brazil) and ST57, ST101, ST1266, and ST366 (Columbia); these STs were not detected in our study [44,48].

All of the 3GC-R-*Ec* isolates except two exhibited resistance to at least three antibiotic classes, with acquired resistances to tetracyclines, quinolones (nalidixic acid and ciprofloxacin), and sulfonamides being the most frequent combination. While the resistances in the majority of the isolates were linked to the acquisition of a specific resistance gene, the high levels of resistance to fluoroquinolones observed in 15 isolates were attributed to known mutations in the quinolone resistance-determining regions (QRDRs) of ParC and GyrA [13]. Of note, other isolates that contained only *aac(6)-Ib-cr* or *qnr* without mutations in the QRDRs of ParC and GyrA exhibited low-level resistance to fluoroquinolones, as has been reported previously [13,53,54]. In addition, the presence of *aac(6)-Ib-cr* and *qnr* genes was recently reported in ESBL-producing ExPEC strains from clinical settings in Cuba [28]. Although it is difficult to determine the driving force for the selection of these genes in bacteria from poultry, their presence may be related to the use of quinolones in poultry production in Cuba [55]. While antibiotics are not used as growth promoters and as prophylactics in poultry in Cuba, some antimicrobials belonging to the fluoroquinolones, tetracyclines, β-lactams, macrolides, aminoglycosides, sulfonamides as well as colistin are approved for use in poultry for the treatment of respiratory and intestinal tract infections [55]. Their use may have contributed to the selection and maintenance of 3GC-R-*Ec* and their resistance genes on plasmids in poultry in Cuba, emphasizing the prudent use of these antibiotic classes in poultry. Plasmids encoding ESBL genes may also carry genes for resistance to other antimicrobial agents, such as aminoglycosides, trimethoprim, sulfonamides, tetracyclines and chloramphenicol [8,56]. The coselection phenomenon in ESBL-producing *E. coli* from aviculture and humans has been suspected as a possible cause for the spread of multiresistant strains [57,58,59]. It is, therefore, strongly recommended to limit the use of antibiotics that are critically important for human health in animals [60,61].

## 4. Materials and Methods

### 4.1. Sampling

**Figure 4:**
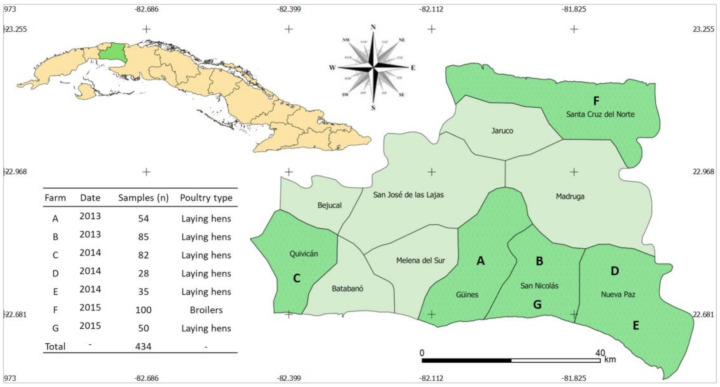
Sampling information (sampling size and year, poultry type) and geographical location of the sampled poultry farms (A–G) in different municipalities of the province of Mayabeque, Cuba.

During the 2013–2015 period, a total of 434 cloacal swab samples were collected from healthy birds from six farms rearing laying hens in cages and one farm producing broilers via floor rearing for meat production. The farms were located in different regions of Mayabeque Province, Cuba. The cloacal samples from 2013 (*n* = 139) and 2014 (*n* = 145) were collected at farms A–B and C–E, respectively, and those from 2015 (*n* = 150) were from farms F–G (Figure 4). The samples were collected by veterinarians according to an approved procedure [62]. Only one sample per animal was taken. The samples were directly placed into 1 mL Tryptone Soy Broth (Oxoid, Basingstoke, UK) medium and kept cool during transport to the laboratory, where they were processed immediately.

### 4.2. Isolation and Identification of 3GC-R-Ec

The inoculated Tryptone Soy Broth (Oxoid) medium was transferred into 5 mL of MacConkey Broth (Oxoid) containing 8 mg/L of ceftazidime (Sigma-Aldrich, Merck, St. Louis, MO, USA) and incubated at 37 °C for 24 h under agitation. Then, a loopful (10 μL) of the mixture was plated onto MacConkey Agar (BioCen, BioCubaFarma, Bejucal, Cuba) supplemented with 8 mg/L of ceftazidime (Sigma-Aldrich) for the screening of 3GCs-R *Enterobacteriaceae* and reincubated overnight. Lactose-positive (pink colonies) were selected and streaked three times on selective MacConkey Agar plates to obtain pure culture. Single pink colonies from each selective plate were streaked onto Tryptone Soy Agar plates containing 5% sheep blood (TSA-SB; Becton Dickinson, Franklin Lakes, NJ, USA) and incubated overnight at 37 °C. The colonies were identified using a matrix-assisted laser desorption/ionization time-of-flight mass spectrometer (MALDI-TOF MS, Microflex LT; Bruker Daltonics, Billerica, MA, USA) and frozen at −80 °C in glycerol stocks.

### 4.3. Rep-PCR-Based Genetic Relatedness and Selection of 3GCs-R-Ec Isolates

All 3GCs-R-*Ec* isolates were submitted to rep-PCR as described previously [63]. A dendrogram was generated using BioNumerics software v.7.6 (Applied Maths, Sint-Martens-Latem, Belgium). The isolates showing more than 90% rep-PCR pattern similarity were considered to belong to the same clonal group generating 39 different lineages (Figure S1). Out of them, thirty-two isolates, which were either nonclonal or which were clonal but came from a different farm, were selected for further antimicrobial susceptibility testing and molecular characterization. Only one isolate per clonal group was selected from the same farm.

### 4.4. Antimicrobial Susceptibility Testing

Measurements of the MICs of antibiotics were obtained by broth microdilution in Mueller-Hinton Broth (Thermo Fisher Scientific, Waltham, MA, USA) using EUVSEC Sensititre^®^ plates (Thermo Fisher Scientific) following the interpretation criteria of the European Committee on Antimicrobial Susceptibility Testing [64] for ampicillin (R > 8 μg/mL), cefotaxime (R > 2 μg/mL), ceftazidime (R > 4 μg/mL), ciprofloxacin (>0.5 µg/mL), chloramphenicol (R > 8 μg/mL), colistin (R > 2 μg/mL), gentamicin (R > 4 μg/mL), meropenem (R > 8 μg/mL), tigecycline (R > 0.5 µg/mL), and trimethoprim (R > 4 μg/mL) and those of the Clinical and Laboratory Standards Institute for nalidixic acid (R ≥ 16 μg/mL), sulfamethoxazole (R ≥ 512 μg/mL) and tetracycline (R ≥ 16 μg/mL) [65]. Multidrug resistance was defined as acquired resistance to three or more different classes of antibiotics.

### 4.5. Detection and Identification of Antibiotic-Resistance Genes

DNA was obtained by incubating half a loop full of bacteria in 400 µL of lysis buffer (0.1 M Tris-HCl pH 8.5, 0.05% Tween 20, and 0.24 mg/mL proteinase K) for 45 min at 60 °C followed by 15 min at 95 °C [43]. Antibiotic resistance and virulence factor genes were detected using AMR08 ArrayStrip microarrays and the Hybridization Plus (+) Kit (Alere Technologies GmbH, Jena, Germany); a signal intensity of 0.1 or higher was considered positive. The presence of the β-lactam resistance genes *bla*_CTX-M-1_, *bla*_CTX-M-15_ and *bla*_LAP-2_ was confirmed by PCR and DNA sequencing [43,66]. The amino acid substitutions in the quinolone resistance-determining regions of GyrA and ParC were detected as described previously [67].

### 4.6. Multilocus Sequence Typing (MLST) and Determination of Phylogenetic Group

MLST was determined using the Achtman scheme and the *E. coli* database at Enterobase (https://enterobase.warwick.ac.uk/) [68]. The minimum spanning tree of MLST was constructed with Bionumerics 7.6 (Applied Maths). The phylogenetic groups of the isolates were determined using the triplex PCR method as previously described [69].

### 4.7. Pulsed-Field Gel Electrophoresis (PFGE)

The genetic similarity of the 3GCs-R-*Ec* isolates was determined by PFGE using XbaI digests and conditions as previously described [43]. The definition of a PFGE cluster was based on a similarity cut-off of 90% (Dice coefficient, represented by unweighted pair-group method with arithmetic mean (UPGMA), 1.5% optimization and 1% tolerance). The dendrogram was constructed with Bionumerics 7.6 (Applied Maths).

### 4.8. PCR-Based Replicon Typing (PBRT) of Plasmids

The incompatibility groups of plasmids were investigated and characterized using the PBRT Kit (DIATHEVA, Cartoceto, Italy) as previously described [29].

### 4.9. Statistical Analysis

Multiple correspondence analysis (MCA) was used to analyze the patterns of association between the characteristics of the isolates (ST, phylogenetic group, genotypic and phenotypic resistance or susceptibility to different antibiotics) and their distributions across the farms. The inertia values were calculated by the standard “Burt matrix” method. The analyses were performed using the statistical software package Statgraphics Centurion v. 16.1.03 (StatPoint Technologies Inc, Warrenton, VA, USA).

## 5. Conclusions

This study demonstrated, for the first time, the presence of 3GC-R and multiresistant *E. coli* in poultry in Cuba, and it revealed some of the genetic characteristics of the isolates. These findings represent a baseline for the establishment of a nation-wide system of surveillance of resistance in food-producing animals and the implementation of strategies for the prudent use of antibiotics in poultry production in Cuba. The results also emphasize the importance of strengthening good hygiene and sanitation practices to prevent the spread of antimicrobial resistance in animals, the environment and humans.

## Data Availability

Data is available within the article as well as in Supplementary Table S1 and Figure S1.

## Supplementary Materials

The following are available online at https://www.mdpi.com/2079-6382/10/2/107/s1, Figure S1: Cluster analysis of rep-PCR fingerprints of the 3rd-generation cephalosporin-resistant *Escherichia coli* (3GC-R-*Ec*) isolated from poultry in the province of Mayabeque, Cuba, Table S1: Minimal inhibitory concentration (MIC) values of 14 antimicrobials for 32 isolates of 3rd-generation cephalosporin-resistant *Escherichia coli* (3GC-R-*Ec*) obtained from poultry in the province of Mayabeque, Cuba.
