# Supplementary material for: Genetic Features of Extended-Spectrum β-Lactamase-Producing Escherichia coli from Poultry in Mayabeque Province, Cuba
Source: Antibiotics (Basel). 2021 Jan 22;10(2):107. doi: 10.3390/antibiotics10020107 (PMC7910960; doi:10.3390/antibiotics10020107)
Supplement: Supplementary file 1 [file antibiotics-10-00107-s001.pdf]

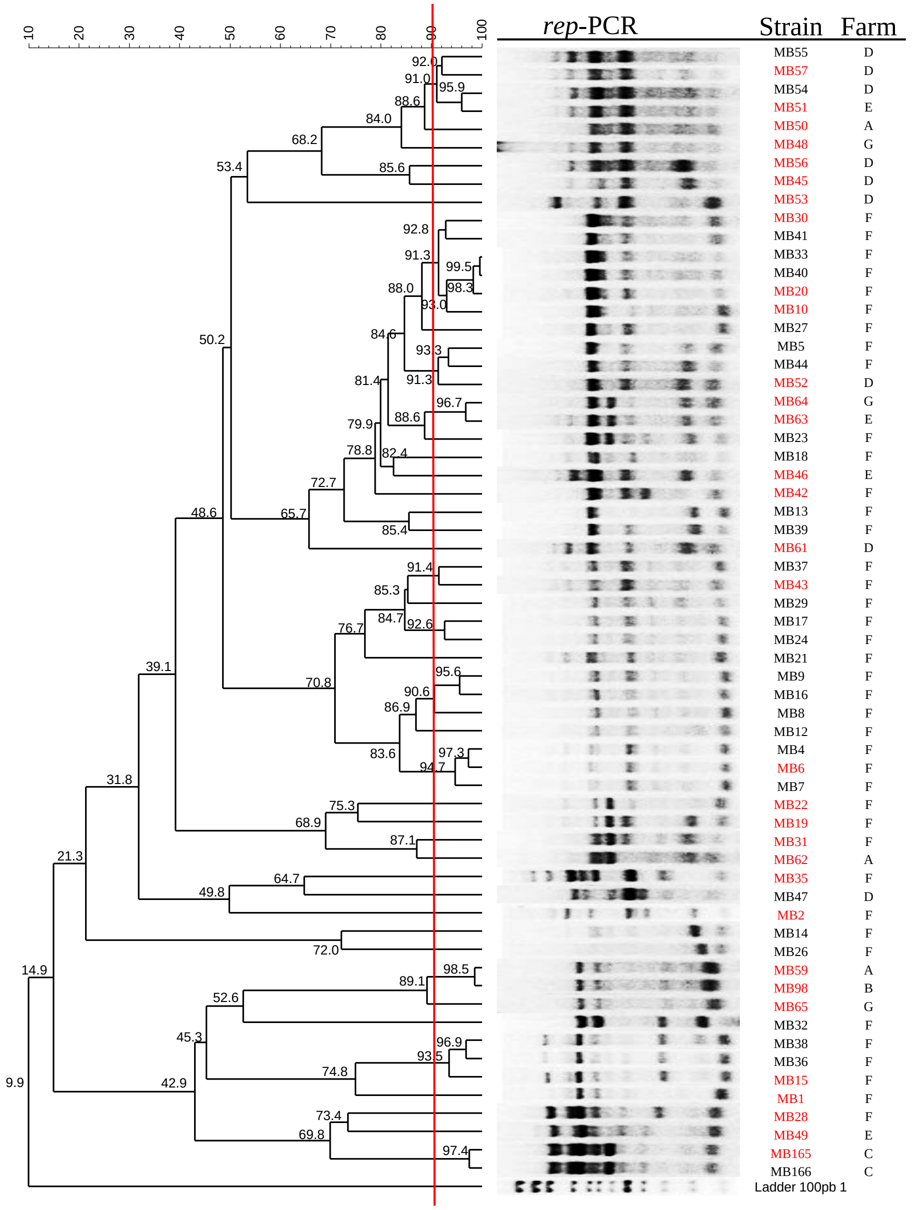

Figure S1. Cluster analysis of *rep*-PCR fingerprints of the 3rd-generation cephalosporin-resistant *Escherichia coli* (3GC-R-*Ec*) isolated from poultry in the province of Mayabeque, Cuba. The clustering of the isolates was based on a similarity cut-off of 90% [Pearson product-moment correlation coefficient (Pearson's correlation), represented by unweighted pair-group method with arithmetic mean (UPGMA), 1% optimization and 0.5% tolerance]. Numbers in red indicate the representative *E. coli* isolates that were selected for molecular typing. The dendrogram was generated using BioNumerics software v.7.6 (Applied Maths, Sint-Martens-Latem, Belgium).

Table S1: Minimal inhibitory concentration (MIC) values of 14 antimicrobials for 32 isolates of 3rd-generation cephalosporin-resistant *Escherichia coli* (3GC-R-*Ec*) obtained from poultry in the province of Mayabeque, Cuba.

**MIC of 14 antibiotics for 32 3GC-R-*Ec* isolates from poultry**																					**Resistance mechanisms and number of strains [n]**
		**Number of strains with MIC (µg/mL) of**																			
**Antimicrobials**	%R	<0.015	0.015	0.03	0.06	0.12	0.25	0.5	1	2	4	8	16	32	64	128	256	512	1024	>1024	
Gentamicin	28							10	11	2			3	6							*aac(6')-Ib-cr* [15]
Sulfamethoxazole	59											4	4	3	2				1	18	*sul1* [5]; *sul2* [12]; *sul3* [5]
Colistin	0								31	1											-
Nalidixic acid	53										4	4	7	1		16					*aac(6')-Ib-cr* [15]; *qnrS* [2]; *qnrB* [16]; GyrA (S83-L [15], D87-N [13], D87-Y [1]); ParC (S80-I [12], E84-G [1])
Ciprofloxacin	47			1	1	2	5	7	1			15									*aac(6')-Ib-cr*[15]; *qnrS*[2]; *qnrB*[16]; GyrA (S83-L[15], D87-N[13], D87-Y[1]); ParC (S80-I[12], E84-G[1])
Ampicillin	100														32						*bla*CTX-M-1 [28]; *bla*CTX-M-15 [4]; *bla*LAP-2[1]
Tetracycline	81									4	2				26						*tet*(A) [16]; *tet*(B) [14]
Cefotaxime	100										32										*bla*CTX-M-1 [28]; *bla*CTX-M-15 [4]; *bla*LAP-2[1]
Ceftazidime	63										12	20									*bla*CTX-M-1 [28]; *bla*CTX-M-15 [4]; *bla*LAP-2[1]
Chloramphenicol	34											21	3	1	3	4					*cmlA1* [3], *floR* [5]
Trimethoprim	56						1	10	2	1				18							*dfrA1* [2]; *dfrA7* [1]; *dfrA12* [4]; *dfrA14* [2]; *dfrA17* [9]
Meropenem	0			31	1																-
Azithromycin	-										7	7	4	6	8						*mph*(A)-*mrx* [16]; *mph*(D)-*mrx* [1]
Tigecycline	0						21	11													-

Antimicrobial resistance genes and functions: *bla*CTX-M, extended-spectrum β-lactamase genes for resistance to third-generation cephalosporins; *aadA4*, aminoglycoside nucleotidyltransferase gene for streptomycin and spectinomycin resistance; *cmlA1*, chloramphenicol efflux gene; *flor*, florfenicol/chloramphenicol resistance gene; *dfrA1*, *dfrA7*, *dfrA12*, *dfrA14*, *dfrA17*, and *drfA19*, dihydrofolate reductase genes for trimethoprim resistance; *tet*(A), *tet*(B), tetracycline efflux gene; *strA*, *strB*, streptomycin phosphotransferase genes; *sul1*, *sul2*, *sul3*, dihydropteroate synthase genes for sulfonamide resistance; *mrx-mph(A), mrx-mph(D)*, macrolide inactivation gene clusters; *aac(6´)-Ib-cr*, aminoglycoside N(6')-acetyltransferase-cr gene for amikacin, kanamycin and quinolone resistance; *qnrB*, *qnrS*, DNA gyrase protection genes for low-level resistance to fluoroquinolones; GyrA (S83-L), (D87-N), (D87-Y) and ParC (S80-I), (E84-G), amino acid substitutions in topoisomerase GyrA and ParC for high-level resistance to fluoroquinolones. The vertical bar indicates the resistance breakpoint. The numbers of susceptible and resistant isolates are indicated in blue and red, respectively. For azithromycin, the number of isolates are indicated in black, since no breakpoints are available for *E. coli*.
